# Factors Associated with the Seroprevalence of Leishmaniasis in Dogs Living around Atlantic Forest Fragments

**DOI:** 10.1371/journal.pone.0104003

**Published:** 2014-08-04

**Authors:** Nelson Henrique de Almeida Curi, Ana Maria de Oliveira Paschoal, Rodrigo Lima Massara, Andreza Pain Marcelino, Adriana Aparecida Ribeiro, Marcelo Passamani, Guilherme Ramos Demétrio, Adriano Garcia Chiarello

**Affiliations:** 1 Postgraduate program in Applied Ecology, Department of Biology, Federal University of Lavras, Lavras, Brazil; 2 Postgraduate program in Ecology, Conservation and Management of Wildlife, Department of Biology, Institute of Biological Sciences, Federal University of Minas Gerais, Belo Horizonte, Brazil; 3 Laboratory of Leishmaniasis, Ezequiel Dias Foundation-FUNED, Belo Horizonte, Brazil; 4 Department of Biology, University of São Paulo, Ribeirão Preto, Brazil; Centro de Pesquisa Rene Rachou/Fundação Oswaldo Cruz (Fiocruz-Minas), Brazil

## Abstract

Canine visceral leishmaniasis is an important zoonosis in Brazil. However, infection patterns are unknown in some scenarios such as rural settlements around Atlantic Forest fragments. Additionally, controversy remains over risk factors, and most identified patterns of infection in dogs have been found in urban areas. We conducted a cross-sectional epidemiological survey to assess the prevalence of leishmaniasis in dogs through three different serological tests, and interviews with owners to assess features of dogs and households around five Atlantic Forest remnants in southeastern Brazil. We used Generalized Linear Mixed Models and Chi-square tests to detect associations between prevalence and variables that might influence *Leishmania* infection, and a nearest neighbor dispersion analysis to assess clustering in the spatial distribution of seropositive dogs. Our findings showed an average prevalence of 20% (ranging from 10 to 32%) in dogs. Nearly 40% (ranging from 22 to 55%) of households had at least one seropositive dog. Some individual traits of dogs (height, sterilization, long fur, age class) were found to positively influence the prevalence, while some had negative influence (weight, body score, presence of ectoparasites). Environmental and management features (number of cats in the households, dogs with free-ranging behavior) also entered models as negative associations with seropositivity. Strong and consistent negative (protective) influences of the presence of chickens and pigs in dog seropositivity were detected. Spatial clustering of cases was detected in only one of the five study sites. The results showed that different risk factors than those found in urban areas may drive the prevalence of canine leishmaniasis in farm/forest interfaces, and that humans and wildlife risk infection in these areas. Domestic dog population limitation by gonadectomy, legal restriction of dog numbers per household and owner education are of the greatest importance for the control of visceral leishmaniasis in rural zones near forest fragments.

## Introduction

Landscape changes such as urbanization and human encroachment are among the main drivers of the alteration of disease dynamics, e.g., the increased or altered prevalence and incidence of disease in humans, domestic animals, and wildlife [1]–[4]. The introduction of exotic domestic species often accompanies human movements during such changes and poses a threat to both wildlife and human health. Since their domestication, pet animals have been closely associated with humans, and dogs (*Canis familiaris*) are the most common and distributed companion animal worldwide [5]–[6]. Unfortunately, this ubiquitous human-dog bond also brings many host species into contact with their pathogens because dogs occupy both natural and human-modified areas and may therefore enhance disease transmission and persistence in humans and wildlife [7]. But because of this close bilateral interaction, domestic dogs may also be used as sentinels of disease for both human and wildlife populations [8]–[10].

In Brazil, there are about 40 million dogs, of which five million are represented by rural dogs. Most of these live unrestricted, exhibiting free-ranging behavior, and move in both urban and natural areas [6]. Accordingly, recent studies have shown that the domestic dog has become increasingly common in several Brazilian protected areas [11]–[12], but the ecological and epidemiological impact of this invasion generally remains unknown. In a study conducted in India, Vanak and Gompper [13] have shown that dogs interfere with the spatial distribution of sympatric native carnivore species. Therefore, they also disturb the spatial distribution of hosts and parasites, affecting disease dynamics and the resulting impact on wildlife and human populations that have contact with these dogs. The contact events and the presence of parasites in domestic dogs indeed increase the risk of disease for both humans and wildlife [7], [14]–[15] and must be investigated if the aim is to minimize risk and to understand the dynamics of the systems into which dogs are introduced and with which they interfere. Human behavior also has the potential to alter parasite dynamics in wildlife-human-domestic animal interfaces [16]. For instance, wild carnivores are more exposed to pathogens in places where they face more frequently their domestic counterparts [15], and dog ownership is itself an important risk factor for human leishmaniasis [14], [17].

Visceral leishmaniasis is a dangerous systemic disease among the most significant zoonosis in Brazil, affecting both dogs and humans. Brazil holds the higher number of cases in South America and is one of the six most affected countries worldwide. The disease is caused by parasites of the species *Leishmania infantum*, whose vectors are phlebotomine sand flies of the genus *Lutzomyia* (Psychodidae) [18]–[20]. The main reservoir of *L*. *infantum* is the domestic dog, although the possible participation of asymptomatic infected persons is currently been suggested [21]–[23]. Other wild mammal species may be infected and may develop clinical signs, but their role as reservoirs remains to be clarified [22], [24]–[26]. One of the few well studied species is the widely distributed and relatively abundant South American wild canid crab-eating fox *Cerdocyon thous*, a host with low infectiveness unable to sustain *Leishmania* cycles without the presence of sympatric dogs [21].

Recent studies have considered the surrounding environment and its relation to the epidemiology of human and canine visceral leishmaniasis (CVL). Their results are mixed, although several interesting patterns have arisen, e.g., the influence of other domestic animals as attractors for the vector, which ultimately produces an increased risk of infection in dogs and humans [27]–[30]. Furthermore, according to a topical review, there is still controversy over risk factors associated with infection in dogs, and surveillance and information is scarce in some areas in Brazil [31]. A recently published paper has identified peridomestic risk factors for both canine and human cutaneous leishmaniasis in an agricultural area of southern Brazil [32].

Visceral leishmaniasis affects mostly poor communities in remote rural areas [19]. However, for CVL, many areas and contexts such as rural settlements around forest fragments and other human-wildlife-domestic animal interface zones have been poorly evaluated. The control and elimination of leishmaniasis is far from realistic in Latin America because it is a zoonosis with a very large domestic reservoir and probably a substantial sylvatic reservoir (though this is a point which still needs further investigation), and the existence of gaps in knowledge and surveillance along with a lack of political involvement [33]. Thus, the goals of this study are to evaluate the seroprevalence of CVL, a neglected but important zoonosis in Brazil, in areas of unknown epidemiological status in the Atlantic Forest domain and to correlate this presence with dog individual traits, animal management and environmental factors. In this way, the patterns of infection detected here can ultimately be targeted or managed by programs for the control of the disease.

## Materials and Methods

### Ethics statement

Sampling and interviewing were performed under consent obtained from the household head or other responsible individual. Licenses from the State Forest Institute – IEF (UC: 080/10, 081/10 and 082/10) and approval from the Ethics Commission on the Use of Animals of the Pontiphical Catholic University of Minas Gerais (CEUA, PUC Minas 037/2010) were obtained prior to the initiation of the field work. Regarding the collection of data from human participants, our project was examined by the Ethics Research Committee (Comitê de Ética em Pesquisa) of the Pontiphical Catholic University of Minas Gerais (PUC-Minas). We did collect some information on the number of people inhabiting the house with the approved consent of the household head. A Consent Term about the confidential character of the records was read to every interviewed person. Animal manipulation procedures adhered to the recommendations from the COBEA (Brazilian College of Animal Experimentation) and the Animal Ethics Committee of FIOCRUZ (Oswaldo Cruz Institute Foundation) of the Brazilian Ministry of Health.

### Study sites

**Figure 1:**
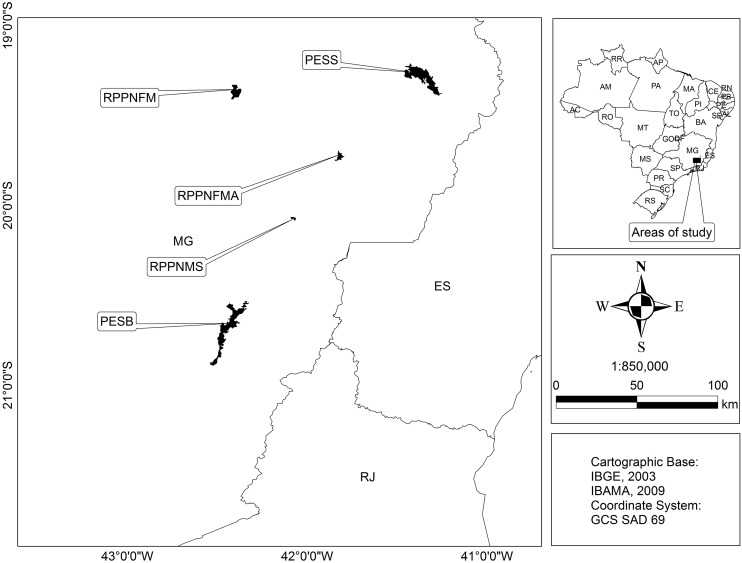
Study areas location in the Atlantic Forest domain, Minas Gerais state, southeastern Brazil.

**Figure 2:**
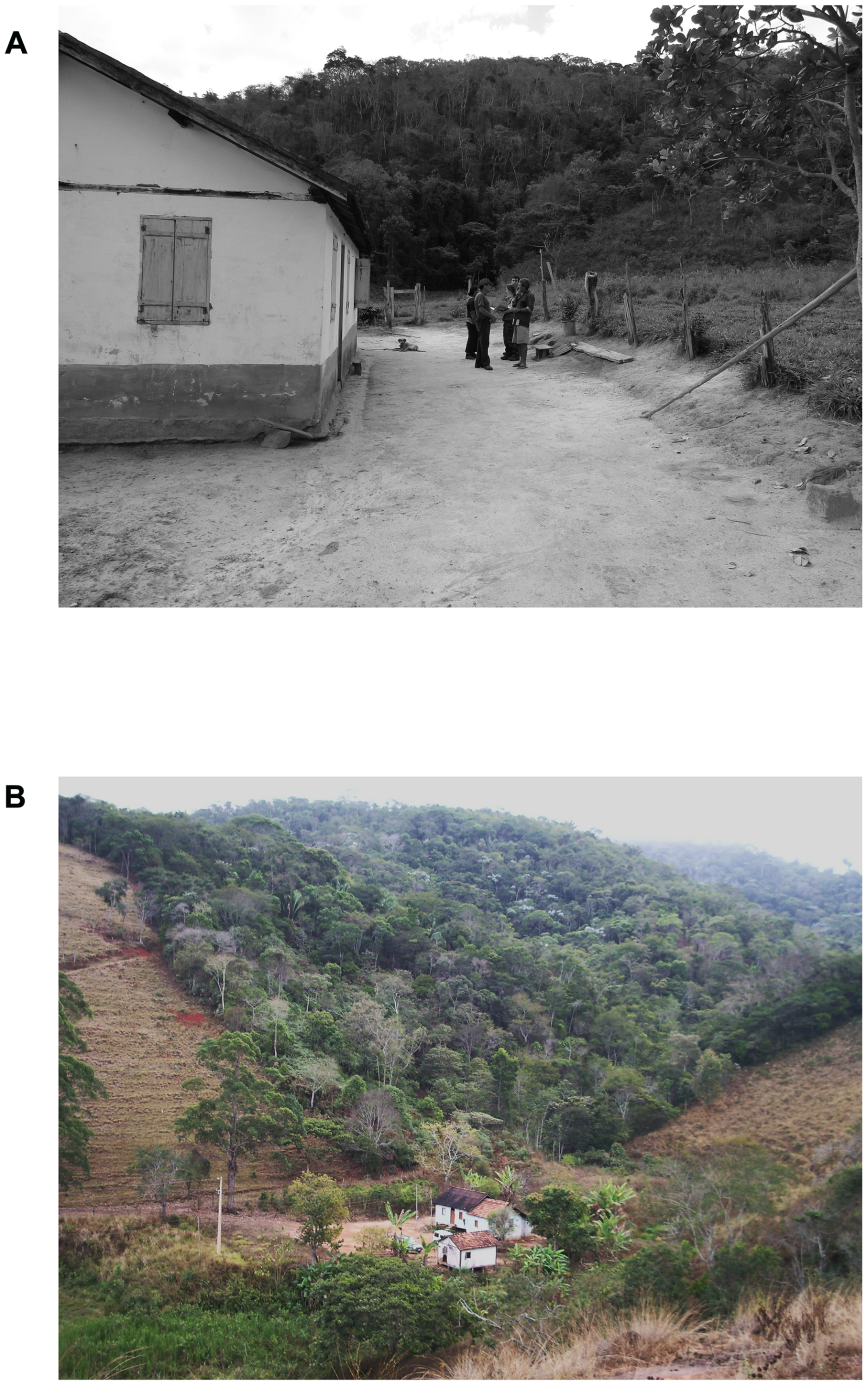
Typical households and peridomestic scenarios of rural areas surrounding Atlantic Forest fragments in Minas Gerais State, southeast Brazil.

Rural settlements surrounding five protected areas in the Atlantic Forest domain of the state of Minas Gerais, southeastern Brazil, were selected for this study. These areas comprise two state parks, Serra do Brigadeiro (PESB, municipality of Araponga) and Sete Salões (PESS, municipality of Santa Rita do Itueto), and three private reserves, Fazenda Macedônia (RPPNFM, municipality of Ipaba), Feliciano Miguel Abdala (RPPNFMA, municipality of Caratinga), and Mata do Sossego (RPPNMS, municipality of Simonésia) (Figure 1, table 1). All of the areas had humans living in their vicinity and various degrees of domestic dog occupancy recorded within their borders [12]. The landscapes around the protected areas are mostly composed of a mosaic of forest borders, small rural properties, their legal reserves and small human settlements. Households were mostly located near forests, water bodies, and had vegetation in their vicinities (Figure 2), which are considered risk factors for *Leishmania* infection [31]. According to the official Brazilian health services, these areas are characterized by an absence of recorded human leishmaniasis cases except for Ipaba municipality, where a few records have been obtained in recent years (Table 1). Several species of the genus *Lutzomyia* occurs at the Atlantic Forest in both peridomiciliary and forest environments [34]–[35]. All households were located near potential breeding sites for the vectors (forested areas, water bodies, peridomiciliary microhabitats and plantations). Sand flies are indeed abundant in human-disturbed open areas such as plantations and secondary forest and homesteads with the presence of dogs [36]. Thus, our sampling sites located in rural/forest interfaces are likely not free of the presence of vector species.

**Table 1:** Epidemiological features of five protected areas in the Atlantic Forest of the state of Minas Gerais, southeastern Brazil.

Study site	Distance fromnearest city (km)	Altitude (m)	Area size (ha)	Transmission status^1^	Human cases^1^/population^2^	Human:dog ratio
RPPNFM	0.3	320	3,343	Sporadic	2/16,708	1.2
PESB	3.3	1,437	15,015	Silent	0/8,152	1.9
PESS	4.7	687	13,370	Silent	0/5,697	1.8
RPPNFMA	10.5	430	1,312	Silent	0/22,242	1.1
RPPNMS	7.7	1,340	392	Silent	0/18,298	2.9
Total	-	-	-	-	2/71,097	1.8 (0.2–8)

^1^Data from 2010–2012 (Brazilian Ministry of Health).

^2^Data from the 2010 census (Brazilian Institute of Geography and Statistics, www.ibge.gov.br).

### Sampling

The study was conducted between January 2011 and August 2012. Overall, 291 dogs older than two months were sampled in 124 rural households located up to two kilometers from protected area boundaries around the study areas, and this was the sole eligibility criteria used for this study. After physical restraint, blood was collected from the jugular vein and a complete clinical examination of the dogs was performed (focusing on clinical alterations of visceral leishmaniasis such as weight loss, skin lesions, nail overgrowth and increased volume of the liver and spleen). A standardized questionnaire survey was administered to the owners. Factors related to animal management and behavior (number of dogs, mobility of dogs, access of dogs to the forest and villages, observed interactions between dogs and wildlife, ectoparasite treatment), the presence of vector attractors in peridomestic dwellings (i.e., other domestic species), number of people and geographic coordinates were recorded for each household. The individual and clinical features of the dogs (sex, age, height, weight, fur type, breed, sterilization, body condition, clinical alterations, and the presence of ectoparasites such as fleas and ticks) were recorded in individual files. Weight was measured with a precision scale (Pesola, 50 kg capacity), and height was measured from the footpad to the top of the scapulae of standing dogs. Body condition of dogs was scored from 0 (extreme emaciation) to 5 (extreme obesity). Refusals to the survey occurred in four cases because the responsible were absent from the households at the time of collection. There were no other refusals, and we believe that the houses that were not surveyed did not affect the overall results.

### Laboratory analysis

Blood samples were allowed to clot for 4 h at room temperature and then centrifuged for serum extraction. Serum samples were initially stored at −20°C, and sent later to be stored at −80°C at Fundação Ezequiel Dias, Belo Horizonte, prior to analysis. Immune enzyme assays (ELISA), indirect immunofluorescence reaction (IFI), and dual path platform immunochromatographic rapid test (DPP) analyses were performed using Biomanguinhos kits (Fiocruz, Manguinhos, Rio de Janeiro, Brazil). These tests are currently used for the diagnosis of CVL in endemic areas by the laboratories of public health [37]–[39]. IFI tests were performed with a cut-off point at the dilution of 1∶40. The ELISA results are expressed in absorbance values and the DPP test provide visual interpretation of seropositivity.

### Statistical analysis

Spearman correlation matrices were built in order to test correlations and assess the level of agreement between the three serological tests, as well as to assess correlation between the ectoparasite presence and previous insecticide treatment in dogs. Dog individual traits, ecological (presence of animals attractive for the sand flies), and management factors (level of dog’s restriction, access to forest and urban areas, and ectoparasite treatment) that may be linked to CVL transmission according to previous literature (see [31] and related papers) were used as explanatory or independent variables for different scenarios of seropositivity (positives for at least one test, ELISA, IFI, and DPP positives, and paired tests) for *Leishmania* in dogs, the binary response (dependent) variables. Households were considered positive if they had at least one seropositive dog. At the individual level, sex, age class (younger or older than 12 months), fur type (fur less than 3 cm long was considered short), sterilization, breed (purebred and mixed bred), and the presence of ectoparasites were used as the independent binary variables. Age, weight, height and body condition were included as quantitative variables. For the households, the continuous variables were the numbers of dogs, people, and cats. The presence of chickens, livestock mammals (cattle, horses and pigs), small pets (e.g. rabbits and birds), whether dogs were kept free or not, the access of dogs to the nearest cities and to the protected areas, whether owners observed interactions with wildlife, and ectoparasite treatment, were included as binary factors. Generalized linear mixed models (GLMMs) adjusted with a binomial distribution for the response data and controlling for households and areas as random effects (all other variables were set as fixed effects in the models), were used to select the most important factors or combinations of factors associated with seropositivity. This type of model is considered suitable for cross-sectional epidemiological studies [40]. The variables were subsequently removed from the complete model (significantly different from a null model) by a backward stepwise approach according to their level of significance, until the difference between subsequent models was significant (p<0.05). Comparisons of prevalence ratios among the study areas, and for binary variables of dog individual traits (gender, sterilization, age class: young (<1 yr) versus adult (>1 yr), pure breed versus mixed breed, short fur versus long fur dogs, presence of ectoparasites), and management and environmental features (mobility, access to forests and villages, presence of other domestic animal species, interactions with wildlife and previous ectoparasitic treatment) were performed with multiple and two proportion Yates-corrected Chi-square tests. We did not applied Chi-square or similar tests with the prevalence ratios of continuous variables to avoid unnecessary data categorization and redundancy with the GLMM tests. A threshold of p<0.05 was used to determine statistical significance. The GLMM tests were run in package lme4 of R software, and the other analyses were performed in BioEstat 5.0 [41]–[42]. To assess spatial clustering of seropositive dogs, we used a nearest neighbor dispersion analysis of dog locations with the software BIOTAS version 2.0a 3.8. We based on the STROBE statement [43] as a guide for the reporting of our observational results.

## Results

The sex ratio of the dogs was 2∶1 (193 males: 98 females), the average age of the dogs was 3.3 yr (ranging from 3 months to 18 yr), and adult dogs (>1 yr old) represented 78% of the total (227/291). Only 8.6% (25/291) of the dogs had long fur, and purebred dogs represented 15.8% (46/291). The mean body condition score was 2.2 (ranging from 0.5 to 3.5). Low body scores (up to 2) were detected in 170 (58.4%) dogs. Ectoparasites (fleas or ticks) were found in 86% of the dogs, and 77% (226/291) were submitted to previous ectoparasite treatment, and infestation were inversely but weakly correlated to previous treatment (r = −0.12; p = 0.032). Only nineteen dogs (6.5%) had been sterilized. The mean number of dogs per household was 2.8 (including dogs that could not be sampled, maximum number = 15). Ninety-five percent (278/291) of dogs were kept without space restriction. The mean number of people was 3.6 per household, with a maximum of eight. Average human to dog ratio was approximately 2∶1. In 80% of the households, the dogs had access to the forest, and they had access to the nearest cities in 36.5% of the households. Chickens were present in 90%, cattle in 55%, horses in 46%, pigs in 38%, cats in 48%, and small pets (rabbits and cage birds) in 14.5% of the households.

There was low correlations between the serological tests used (r = 0.42, p<0.0001 for IFI and ELISA; r = 0.23, p<0.0001 for IFI and DDP; r = 0.05, p = 0.3131 for ELISA and DPP). The ELISA test revealed 13.7% (40/291) of positive samples (39% of positive samples had absorbance values above the cut-off point, including those from symptomatic dogs). Only 9.6% (28/291) of the dogs were seropositive for *Leishmania* sp. according to the IFI test. In the DPP test, eleven samples (3.8%) were positive. When tests were combined, 5.5% of the samples (16/291) were positive for ELISA and IFI. Three samples (1%) were positive for ELISA and DPP. Five samples (1.7%) tested positive for IFI and DPP, and only three samples (1%) were positive for all tests. Because of the low level of agreement among the diagnostic methods used, we calculated prevalence data based on the number of dogs seropositive for at least one test.

Overall seropositivity was 19.9% (58/291). Ten of the 58 positive dogs (17%) were symptomatic for leishmaniasis, showing clinical signs such as weight loss, skin lesions, and nail overgrowth. Forty eight of 124 (38.7%) households had at least one seropositive dog. If the protected areas were considered separately, seroprevalence ranged from 10 to 32% in dogs and from 22 to 55% in households, with significant differences in the prevalence between the areas. Dog and household prevalence were significantly higher in PESB and RPPNMS (Table 2). Differences in prevalence ratios regarding binary variables were detected by the Chi-square tests for the cohabitation of dogs with chickens and pigs (Table 3).

**Table 2:** Seroprevalence of canine leishmaniasis in rural dogs sampled around five protected areas of the Atlantic Forest.

Study site	Number of dogs	Number sampled (%)	Dogs/house	Dog prevalence	P value	Household prevalence	P value
RPPNFM	98	84 (85)	3.9	13.1% (11/84)	<0.0001	40% (10/25)	0.4233
PESB	86	67 (77)	2.7	32.8% (22/67)	0.0072	54.8% (17/31)	0.4723
PESS	53	48 (90)	2.1	14.6% (7/48)	<0.0001	24% (6/25)	0.0163
RPPNFMA	60	50 (83)	3.3	10% (5/50)	<0.0001	22.2% (4/18)	0.0184
RPPNMS	49	42 (85)	1.9	30.9% (13/42)	0.0136	44% (11/25)	0.6889
Total	346	291 (84)	2.8 (1–15)	19.9% (58/291)	<0.0001	38.7% (48/124)	0.0270

**Table 3:** Prevalence ratios for *Leishmania* seropositive dogs (for at least one test) in rural areas around Atlantic Forest fragments, and Chi-square tests results for binary variables.

Variable	Category	Number	Positives	Prevalence ratio	Z	P value
Gender	Males	193	37	19.2		
	Females	98	21	21.4	0.45	0.64
Sterilized	Yes	19	6	31.6		
	No	272	52	19.1	1.32	0.18
Breed	Mixed bred	245	51	20.8		
	Purebred	46	7	15.2	0.87	0.38
Hair	Short	266	52	19.5		
	Long	25	6	24.0	0.53	0.59
Age class	Young	64	9	14.1		
	Adult	227	49	21.6	−1.33	0.18
Ectoparasites	Yes	255	50	19.6		
	No	36	8	22.2	0.36	0.71
Mobility	Free	278	54	19.4		
	Restrained	13	4	30.8	1	0.31
Access to forest	Yes	239	46	19.2		
	No	52	12	23.1	0.62	0.53
Access to villages	Yes	75	11	14.7		
	No	216	47	21.8	−1.32	0.18
Presence of chickens	Yes	271	46	17.0		
	No	20	12	60.0	4.64	<0.0001
Presence of cattle	Yes	180	30	16.7		
	No	111	28	25.2	1.77	0.07
Presence of horses	Yes	153	27	17.6		
	No	138	31	22.5	−1.02	0.3
Presence of pigs	Yes	155	19	12.3		
	No	136	39	28.7	−3.49	0.0005
Presence of small pets*	Yes	59	9	15.3		
	No	232	49	21.1	1	0.31
Interaction with wildlife	Yes	161	32	19.9		
	No	130	26	20.0	−0.02	0.97
Ectoparasite treatment	Yes	226	41	18.1		
	No	65	17	26.2	−1.42	0.15

*Rabbits and cage birds.

The results of the GLMM modeling are summarized in table 4. Models for four of eight possible scenarios (DPP, DPP+IFI, DPP+ELISA, DPP+ELISA+IFI) could not be built due to the small number of positive outputs. In the four viable final models, eleven of 23 entered variables remained in at least one model. The presence of pigs entered all models as a negative association, while the presence of chickens featured in three models, also negatively associated with prevalence. Weight and body score entered two models with negative relationships to infection. The presence of ectoparasites, number of cats per household and mobility of dogs figured in one of the four final models showing negative relationships with seropositivity.

**Table 4:** Best supported GLMMs analyzing associations for leishmaniasis-seropositive rural dogs living around Atlantic Forest fragments.

Scenario/Variables	Estimate (SE*)	Z	P value
**+ in at least one test**			
Sterilized	1.196 (0.569)	2.1	0.03558
Weight	−0.130 (0.044)	−2.9	0.00341
Height	0.139 (0.036)	3.7	0.00016
Presence of chickens	−1.778 (0.530)	−3.3	0.00079
Presence of pigs	−1.084(0.347)	−3.1	0.001804
**+** **ELISA**			
Height	0.043 (0.020)	2.09	0.03663
Presence of chickens	−1.411 (0.557)	−2.5	0.01136
Presence of pigs	−1.144 (0.417)	−2.7	0.00616
**+ IFI**			
Sterilized	2.294 (0.766)	2.9	0.002739
Body score	−1.132 (0.501)	−2.2	0.024009
Weight	−0.205 (0.083)	−2.4	0.013545
Height	0.142 (0.052)	2.7	0.006397
Presence of ectoparasites	−1.582 (0.659)	−2.4	0.016469
Number of cats	−0.453 (0.218)	−2.07	0.038373
Mobility of dogs	−2.976 (0.823)	−3.6	0.000301
Presence of pigs	−0.992 (0.480)	−2.06	0.039026
**+ ELISA/+ IFI**			
Sterilized	1.307 (0.618)	2.1	0.034550
Long fur	1.375 (0.574)	2.4	0.016681
Age class	1.130 (0.597)	1.89	0.058377
Body score	−0.824 (0.344)	−2.4	0.016719
Height	0.048 (0.020)	2.4	0.015223
Presence of chickens	−1.919 (0.546)	−3.5	0.000442
Presence of pigs	−1.343 (0.384)	−3.5	0.000481

*Standard error.

Height of dogs appeared in all models as a positive association with CVL. Sterilization was positively associated with infection in three of four scenarios. Long fur entered one model with a positive association. Age class was positively associated with infection in one model. The correlation matrices provided contained no value above 0.6, thus no colinearity was found that would have prevented the variables to be included in the same model.

Spatial clustering of seropositive dogs was detected only in PESB (Table 5), and seropositive dogs were randomly or uniformly distributed in the other four sites.

**Table 5:** Nearest neighbor dispersion analysis results for leishmaniasis seropositive rural dogs around five protected fragments of the Atlantic Forest in the State of Minas Gerais, Brazil.

Study site	Mean distance betweenseropositive dogs (m)	Distance standard deviation	Z score	Spatial pattern
RPPNFM	951.4	166.2	0.12	Random
PESB	160.8	47.5	−4.48	Clustered
PESS	1351.7	162.5	3.91	Uniform
RPPNFMA	874.3	112.4	4.04	Uniform
RPPNMS	298.6	64.7	−1.38	Random

## Discussion

Because the dog is the primary reservoir and the infection in dogs generally precedes human cases [22], more attention should be given to the disease in dogs wherever they occur, i.e., all human-occupied areas. Even though relatively few humans live in our study areas and have access to these dogs, and the ecological impact of leishmaniasis may be greater than the public health impact, rural families’ welfare should never be neglected. Additionally, there is ecotourism activity inside and around parks, and human encroachment is ongoing at these sites. Consequently, dogs may be useful as sentinels for zoonotic leishmaniasis in areas with uncertain epidemiological status, and efforts to reveal their patterns of infection are of the highest importance for control and prevention.

We acknowledge that the low accuracy of the serological tests used is a limitation of our study and without a molecular test is not possible to rule out cross-reactions with other protozoans, such as *Trypanosoma* sp., in a proportion of dogs sampled. The same serum samples were tested for *Babesia canis* (Curi et al., unpublished data), and only four (1.3%) were positive for both *Leishmania* and *Babesia*. Therefore, the occurrence of this cross reaction may be considered low or nonexistent in this study. Instead, coinfection by both agents is possible. Our results show a low level of agreement between the serological tests used which may be related, among other factors, to the relatively low indirectly estimated (through ELISA) antibody concentrations detected in most samples. Other studies have reported discrepancies in serologic tests, such as differences in sensitivity and specificity [37]. This is of great concern because tests such as ELISA and DPP are currently employed for epidemiological screening and control of CVL in Brazil [38]–[39], and such inconsistency may hamper any research or control efforts. Therefore, our strategy to use concomitantly different serologic tests is recommended, preferably along with molecular diagnostic methods [44].

Many studies have identified risk factors for zoonotic human and CVL. However, most studies on dogs were primarily concerned with urban zones [28]–[32], [44]–[45], [47]. In our study, seven individual traits of dogs were associated with seropositivity. Height was positively associated with seropositivity in all four models. This factor is possibly linked to a target size effect or differences in heat and CO_2_ irradiation between small and large sized dogs, enhancing the finding of larger hosts by the vectors. Weight and body score were negatively associated in two scenarios of seropositivity, and this can be explained by the fact that low body condition animals may have impaired immune function and higher susceptibility to infection. However, dog size was not associated with infection in previous studies [31].

The literature shows that ectoparasites may be positively, negatively or neutrally associated with dog infection [31]. However, despite some controversy, other authors claim that ticks may be able to transmit the parasite [22], [46]. In our study, the presence of ectoparasites in dogs has entered one final model, but with a negative association with seropositivity. This finding do not corroborate with studies from urban areas [31], but the work of Dantas-Torres and colleagues [45] with dogs from a rural community in northeastern Brazil have showed that ticks are not relevant as vectors of *Leishmania*. Our analysis revealed a weak negative correlation between the presence of ectoparasites and previous ectoparasite treatment, meaning that this intervention has been ineffectively performed in the study areas, and is probably either ineffective against sand flies.

Surprisingly, long fur was positively associated with dog seropositivity in our study by one of the models, because, according to the literature, short fur is considered as a strong predictor of canine leishmaniasis infection in Brazilian cities [28], [31], [47]. However this relationship did not hold in our data set. Possibly, the lower densities of rural dogs when compared to urban dogs [6] balance the detectability of shorthaired and longhaired dogs by sand flies. Thus, control measures in rural zones should not target any particular dog phenotype, contrary to the focus on shorthaired dogs proposed for urban populations [31].

Dogs older than one year were more likely to be infected, according to one GLMM scenario. Conversely, age did not enter the models and there was no difference in prevalence between young and adult dogs according to the Chi-square tests. Thus, we believe that age is not a strong predictor for *Leishmania* infection and dogs of all ages may be reservoirs in the study areas, and this is in general agreement with previous literature [31].

Sterilized dogs were found to be seropositive more frequently according to three scenarios. This is expected since gonadectomized dogs tend to roam or escape less and spend more time quiet [48]–[49] being more easily found by the vectors. Conversely, this would depend very much on sand fly density at different sites and peak times of sand fly feeding and of canine resting habits, since sand flies could easily feed on immobile dogs whether they sometimes roam or not.

Four other significant variables linked to dog management (dogs kept free) and vector attractiveness (presence of chickens, pigs and number of cats) entered final models as negatively associated with seropositivity. In the same way as aforementioned about gonadectomized dogs, free-roaming dogs are less sedentary and more difficult targets to vectors, whilst dogs living in restrict spaces spend more time quiet being more easily found, bitten and infected in these rural scenarios. Additionally, the negative association with dog mobility in one of the models indicates that being kept near a human dwelling is associated with increased risk for dog infection. However, in the review of Belo and coauthors [31] is mentioned that the general relationship is the inverse. Perhaps the detectability of dogs by the sand flies varies in some ways between cities and rural areas. Moreover, a purely peridomestic cycle of CVL may be happening in these scenarios, and warrants interesting future investigation.

Negative associations of dog seropositivity and the presence of chickens and pigs were revealed both by the GLMM models and the Chi-square tests. The strongly negative association between positive dogs and the presence of pigs in the households do not agree with most of the past findings. Previous studies have highlighted the presence of large domestic mammals as a positive influence on infection rates in dogs and humans [28]–[29], [31], [50]. Our data show that in these rural sites, the presence of large mammalian livestock (cattle and horses) did not influenced *Leishmania* seroprevalence in dogs, but the presence of pigs may be diverting sand fly bites away from dogs, and then reducing their infection rates. The pig is one of the preferred species as blood sources for the phlebotomines [51], but is apparently an incompetent reservoir [52]. This may facilitate the pig’s zooprophylactic effect against CVL in rural zones, what seemingly happened in our case.

The negative association between the presence of chickens and seroprevalence reveals another evidence of the protective effect of some domestic species against leishmaniasis. This result is also quite controversial because some studies have also identified chickens as attractors for sand flies, implying that the presence of chickens ultimately produces increased infection rates in dogs and humans [27], [28]. Nonetheless, a recent review of risk factors for visceral leishmaniasis in Brazil shows both positive and negative associations of chickens for canine infection [31]. Our results are pointed at the same direction that those aforementioned for pigs. Because chickens are the preferred vertebrate target for the vectors [27], [53] but not suitable hosts for *Leishmania* parasites [54], they also divert the attention of the vectors from the dogs, thus reducing the bite rates and, consequently, the infection rates in dogs. The role of chickens as food sources, vector attractors, and zooprophylactic agents for leishmaniasis has previously been discussed [27], [54]–[55], but only in the context of human infection. The number of cats followed the same pattern, being negatively associated with dog seropositivity (more cats per household are associated with less positive dogs). Cats have been found to be infected with *Leishmania*, can infect sand flies, but do not seem to develop high parasite burdens [22], and may also turn infection away from dogs when in high numbers and densities. Animal sheds and animals on which sand flies feed can increase sand fly density [36] but may also decrease infection prevalence and feeding on dogs and humans, so that the net impact on VL transmission depends on the balance of these outcomes. In our rural context, the balance appears to be favoring a zooprophylactic function of domestic fowl, swine and cats against CVL.

Since there was weak evidence of spatial clustering of seropositive dogs (exclusively for one study site), we believe that the disease is not being maintained in focal points throughout the study areas. Thus, control efforts must be equally employed and cover all properties in these scenarios. One possible explanation for the clustering at PESB is that its higher altitude and the steeper topography drives most human settlements to be located at some of the few valleys and flat areas in the region, resulting in spatial aggregation of households, and consequently, of their dogs.

The Atlantic Forest is a highly diverse and fragmented ecosystem located at the most developed region in Brazil [56]. Therefore, a strong presence of drivers of the dynamic alterations of disease, such as anthropogenic environmental change and increased contact between humans, wildlife, and domestic animals, is expected [4]. However, although governmental prevention programs exist for rural areas, interface areas such as rural zones around forest fragments have received little scientific or government attention in terms of health issues. Our findings show that the study areas should be considered endemic for canine leishmaniasis and that despite the recent trend toward urbanization of the disease [57], it is advisable that government health agencies return to look at rural zones beyond Brazilian urban areas if the aim is to widely control zoonotic leishmaniasis and other tropical diseases. Specifically, in our case, the study areas deserve more attention and thorough investigation through surveys of leishmaniasis in humans, reservoir dogs, wildlife and vectors. Additionally, higher prevalence areas such as PESB and RPPNMS should be prioritized by control programs. The Brazilian visceral leishmaniasis control program should expand the focus to embrace rural and ecosystem health in a holistic view of the problem, and the data presented here should be used as a reference for research and intervention in Brazilian human/wildlife interface areas.

Habitat loss and fragmentation and the subsequent decrease in biodiversity may cause, among many other effects, alterations in parasite ecology that result in increased rates of infection in wildlife [58]–[60]. Although we have no data on wildlife prevalence, the scenario of infected dogs living around and actually entering important biodiversity sites such as Atlantic Forest remnants [12] raises concerns about possible transmission to and from wild animals. Wild mammals can develop clinical signs of leishmaniasis, especially in stressful situations such as captivity [25], and the prevalence of the disease in many captive and free-ranging populations has been reported [22], [24]–[26]. Therefore, the presence of infected reservoir dog populations around small forest fragments under strong human pressure may warrant persistence, circulation, and the possible, yet unknown, deleterious effects of leishmaniasis on the health and fitness of wild animals. Control programs should primarily involve a reduction in the dog population size and density, e.g., by sterilization (not culling), owner education, and legally limiting the number of dogs per rural household in settlements close to wildlife refuges and by restricting the access of dogs to protected areas, thus reducing the probability of disease transmission to and from humans and wildlife. Other measures that reduce attractiveness for sand flies, e.g. application of insecticides and keeping zooprophylactic species such as pigs or chickens around the house may be also recommendable in rural areas. Of course, the latter needs more investigation to detect general patterns before being adopted. Cats are especially not recommended because they cause great damage to wildlife species [61].

Finally, the results presented here suggest another important reason for controlling and monitoring dog populations around protected areas: the risk of visceral leishmaniasis for humans and wildlife. Our findings also highlight the need for additional surveys to detect epidemiological patterns of leishmaniasis in Brazilian rural zones, especially around wildlife-rich protected areas. Another noteworthy aspect of the results is the difference between the profile of risk factors and the results of most previous studies from urban areas. These differences are crucial for planning thoughtful and effective management initiatives that will protect the interdependent health of humans, domestic animals, and wildlife.
